# The Effects of Low Concentrations and Long-Term Contamination by Sodium Dodecyl Sulfate on the Structure and Function of Bacterial Communities in the Lake–Terrestrial Ecotone

**DOI:** 10.3390/microorganisms12112330

**Published:** 2024-11-15

**Authors:** Lingquan Zeng, Qi Zhu, Chunhua Li, Chun Ye

**Affiliations:** National Engineering Laboratory for Lake Pollution Control and Ecological Restoration, State Environmental Protection Key Laboratory for Lake Pollution Control, Chinese Research Academy of Environmental Sciences, Beijing 100012, China; cenglingquan22@mails.ucas.ac.cn (L.Z.); lich@craes.org.cn (C.L.)

**Keywords:** lake–terrestrial ecotone, bacterial communities, sodium dodecyl sulfate, long-term stress, gene function

## Abstract

Due to the growing focus on daily hygiene practices, sodium dodecyl sulfate (SDS), a widely used surfactant, is increasingly found in domestic sewage and rainfall runoff. Upon entering the lake–terrestrial ecotone, SDS affects the composition, abundance, and functional capacity of soil bacterial communities due to its bacteriostatic properties. To investigate the effects of long-term discharge of sewage containing low concentrations of SDS on microorganisms in the lake–terrestrial ecotone, alterations in bacterial community structure, functional genes, and biomass were examined using a simulated continuous pollutant input. The results indicated the following: (1) The degradation rate of sodium dodecyl sulfate (SDS) by soil microorganisms in the lake–terrestrial ecotone under long-term and low concentrations of SDS stress ranged from 11 to 16 mg/kg·d. (2) The effects of low concentrations and long-term SDS stress on bacterial community structure and gene function in the lake–terrestrial ecotone differed significantly from those of short-term pollution. The damage to microbial-promoted material cycling in the lake–terrestrial ecotone was more severe; however, the proliferation of pathogenic bacteria remained continuously suppressed. (3) Soil bacteria in the lake–terrestrial ecotone responded to the stress of long-term and low concentrations of SDS primarily by enhancing chemotaxis and tolerance.

## 1. Introduction

Sodium dodecyl sulfate (SDS), a water-soluble anionic surfactant, possesses bacteriostatic properties [1]. It is extensively employed in both daily life and industrial applications due to its superior decontamination, emulsification, and foaming capabilities, representing 25~30% of the global synthetic surfactant market [2]. Since the outbreak of the novel coronavirus disease (COVID-19) pandemic, the consumption of personal care products with disinfectant properties has steadily increased [3]. For instance, in China, the total output of surfactants in 2022 was 4.262 million tons, a 3.6% increase from the previous year, including 1.718 million tons of anionic surfactants. The ecological impacts of significant quantities of SDS entering natural water bodies, such as rivers and lakes, through domestic sewage and rainfall runoff, have become more severe than ever before.

Since SDS is biodegradable under aerobic conditions, its biodegradability may obscure its environmental risks. Studies have shown that SDS exerts a strong inhibitory effect on microbial activity in the environment and functions as a broad-spectrum organic fungicide [4]. It can alter the permeability of cell membranes, causing the leakage of cellular contents, and it also interferes with enzyme activity by binding to enzymes [5]. Zhou et al. [6] examined the effects and mechanisms of SDS in mitigating substrate clogging in constructed wetlands and found that SDS inhibited bacterial biofilm formation, with SDS dosing leading to a reduction in microbial diversity within the substrate. Gill et al. [7] studied the effects of synthetic and biological surfactants on freshwater biofilm community composition and metabolic activity, and found that SDS reduces microbial diversity in freshwater biofilms, also altering biofilm metabolism. Zheng et al. [8] found that differences in the chemical structures of surfactants (including the charge group) led to divergent metabolic pathways for biodegradation, which shaped biofilm communities. Following the addition of SDS, the structure of the microbial community changed significantly. The relative abundance of *P. knackmussi*, *D. ginsengisoli*, and *P. nitroreducens* decreased from 22% in the attached biofilm to 0.9% in the suspended biomass. The environmental risk posed by SDS is such that when large quantities of SDS enter rivers and lakes through rainwater, it can significantly impact microorganisms in both soil and aquatic ecosystems. Wang Xiuduo et al. [9] studied rainfall runoff pollution in urban areas of Tianjin and found that the concentration of anionic surfactants in runoff from commercial, residential, and cultural areas ranged from 0.808 to 25.640 mg/L. After rainfall, the concentration of anionic surfactants in the river channel increased significantly, from 0.115 mg/L to 1.082 mg/L. The lake–terrestrial ecotone, an inevitable pathway for pollutants to enter lakes, is also a highly active area for microorganisms, performing the ecological function of intercepting and purifying pollutants [10]. When sewage containing SDS enters the lake–terrestrial ecotone, most of the SDS adsorbs to the soil surface, inhibiting soil microbial activity. Although more than 90% of SDS decomposes within 96 h, it still causes a decrease in the nitrogen cycling function of soil microorganisms in the lake–terrestrial ecotone and an increase in human pathogens [11]. A large-scale simulation experiment demonstrated that even low concentrations of SDS significantly altered soil microbial species and functions in the lake–terrestrial ecotone, with this effect persisting for more than 14 days after SDS was completely degraded [12].

Currently, research on the interaction of SDS with microorganisms in the lake–terrestrial ecotone primarily focuses on simulating scenarios where rainfall runoff transports SDS into the lake–terrestrial ecotone. In these conditions, the initial concentration of SDS is elevated but gradually decreases due to microbial degradation. As the concentration of SDS fluctuates, the structure of the soil bacterial community also shifts until the SDS is fully degraded. In a different scenario, the continuous input of sewage into the lake–terrestrial ecotone maintains the concentration of SDS at a stable level over an extended period, ultimately causing soil microorganisms to develop a community structure adapted to the contaminated environment. This scenario is frequently observed near the outfall of sewage treatment plants. The processes of variation in soil bacterial species and functions differ significantly between these two scenarios. Thus, it is necessary to design a dedicated experimental apparatus to assess the structure, function, and degradation rate of soil microorganisms in the lake–terrestrial ecotone, as well as their capacity to degrade SDS under continuous low-concentration input.

This study employs a specialized ecological simulation pool and continuous infiltration device to investigate the long-term effects of low-concentration SDS stress on soil microbial characteristics. Under varying conditions in the riparian zone, the rates of microbial degradation of SDS were measured, revealing differences in the species composition and functional roles of soil microorganisms before and after SDS contamination. Notably, previous studies indicated that the rates of physical and chemical decomposition/transformation of SDS remained stable compared to biological degradation, contributing minimally to the overall removal of SDS. Consequently, this experiment has eliminated the influence of these factors. This research will facilitate a more precise assessment of the environmental impacts of PPCPs, specifically represented by SDS, and will provide theoretical support for the regulation of the usage and discharge of PPCPs. Furthermore, it holds considerable theoretical significance for the ecological protection and management of riparian zones.

## 2. Experimental Section

### 2.1. Experimental Materials

**Figure 1:**
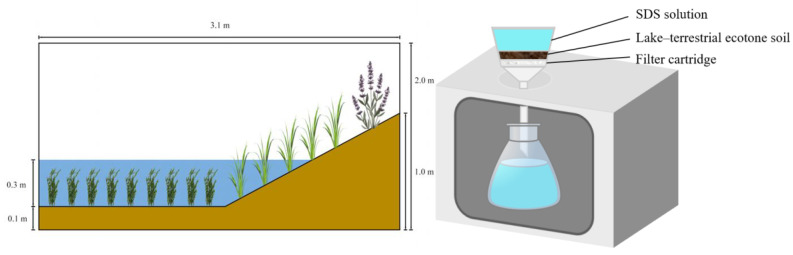

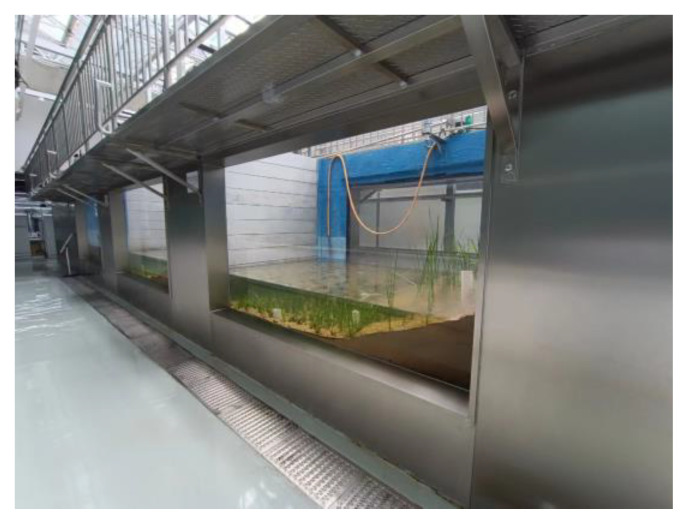
Image of sodium dodecyl sulfate (SDS) input to lake–terrestrial ecotones, experimental and indoor simulation device.

A dedicated lake–terrestrial ecotone ecological simulation pool–continuous infiltration device (Figure 1) was employed to simulate a lake–terrestrial ecotone environment for these experiments. A large-scale simulation pool located within the lake–terrestrial ecotone at the Chinese Research Academy of Environmental Sciences provided the soil for the experiment. The simulation pool had dimensions of 3.02 m in length, 2.60 m in width (with an effective width of 2.10 m), and 2.50 m in height (with an effective water depth of 2.00 m). The interior of the pool was coated with blue epoxy resin for waterproofing and corrosion resistance, and acrylic windows (2.82 m × 1.62 m) were installed on both sides. The facility is computerized to adjust water levels, water quality, light intensity, rainfall, and other factors to closely replicate real environmental conditions along the lake–terrestrial ecotone.

Three simulation pools, numbered from TA to TC, were planted with various species and densities of aquatic plants (see Table 1 for detailed design) to simulate different states of the lake–terrestrial ecotone. The continuous infiltration device consists of a stand, a glass sand core funnel, and a conical flask, with the stand made of a transparent acrylic sheet. The sand core funnel specifications include a volume of 250 mL and a filter plate pore size of 50~70 μm. A low-concentration SDS solution was prepared for the infiltration experiments, simulating long-term and low concentrations input of SDS to the lake–terrestrial ecotone in a gas phase, water phase, and soil phase combination.

### 2.2. Experimental Methods

About 150 g of soil was collected from each of the three large simulation pools in the lake–terrestrial ecotone (Figure 1) and placed into the sand core funnel of the simulation device (Figure 1). During the experiment, 200 mL of SDS solution, at a concentration of 6 mg/L, was added to the funnel every 24 h to ensure continuous infiltration of the SDS solution into the soil. Three replicates were established for each experimental group. Samples of the solution in the funnel, samples from the soil, and the solution in the conical flask were collected every 24 h to measure the concentration of SDS. Additionally, 16S rRNA high-throughput sequencing was employed to assess the relative abundance of bacterial species and functional genes in the soil before the start (0 d) and at the conclusion (10 d) of the experiment, with the soil samples labeled A1,B1,C1 and A2, B2,C2, respectively. Simultaneously, changes in soil microbial biomass carbon (MBC) and soil microbial biomass nitrogen (MBN) were measured in the soil on 0 d and 10 d.

#### 2.2.1. Determination of SDS

Water and soil samples were collected from the simulator, 0.5% of methylene blue solution was added, and SDS was extracted using chloroform. The samples were centrifuged at 4000 r/min for 5 min in a benchtop centrifuge (Pico-21, Thermo Fisher, Waltham, MA, USA), and absorbance was measured at 650 nm using a spectrophotometer (Evolution 300, Thermo Fisher, Waltham, MA, USA) [13].

#### 2.2.2. Determination of Soil Microbial Biomass Carbon (MBC) and Nitrogen (MBN)

The chloroform fumigation–potassium sulfate leaching–TOC instrument method was used to determine microbial biomass carbon (MBC) [14]. A total of 20 g of fresh soil samples were weighed and placed in a 100 mL beaker. A separate beaker containing 60 mL of non-alcoholic chloroform (with zeolite) was also placed into a vacuum desiccator, which contained a small amount of water and NaOH solution (1 mol/L) at the bottom. The desiccator was sealed and pumped with a vacuum pump until the chloroform boiled for 2 min. The desiccator valve was then closed, and it was placed in an incubator at 25 °C for 24 h. Afterward, the chloroform and NaOH at the bottom of the desiccator were removed, and the system was vacuumed for 15~30 min to eliminate any remaining chloroform adsorbed by the soil. Then, 0.5 mol·L^−1^ K_2_SO_4_ solution was added, shaken at 200 r·min^−1^ for 30 min, and then quickly filtered through medium-speed filter paper. The filtrate was either measured immediately or stored at −15 °C, and recorded as MBC_A_. At the start of fumigation, an equal amount of soil sample was taken and subjected to the same leaching method using 0.5 mol·L^−1^ K_2_SO_4_ solution, recorded as MBC_B_. The leaching solution was analyzed using a TOC analyzer (Elemantar: Vario TOC CUBE, Langenselbold, Germany, Europe). MBC = (MBC_A_ − MBC_B_)/0.45, mg·kg^−1^.

The chloroform fumigation–potassium sulfate leaching–flow analyzer method was used for the determination of microbial biomass nitrogen (MBN) [15]. Soil samples fumigated with chloroform were leached with a 0.5 mol·L^−1^ K_2_SO_4_ solution, and the leachate was processed using the Kjeldahl method [14] and subsequently analyzed using a flow analyzer (recorded as MBN_A_). Unfumigated soil samples were also leached with 0.5 mol·L^−1^ K_2_SO_4_ solution, and the same method was applied to a blank sample for comparison (recorded as MBN_B_). MBN = (MBN_A_ − MBN_B_)/0.45, mg·kg^−1^.

#### 2.2.3. 16S r-RNA Sequencing

##### DNA Extraction, Amplification

Total community genomic DNA extraction was performed using a E.Z.N.A.Soil DNA Kit (Omega, M5635-02, Knoxville, TN, USA),following the manufacturer’s instructions. We measured the concentration of the DNA using a Qubit 4.0 (Thermo, Waltham, MA, USA) to ensure that adequate amounts of high-quality genomic DNA had been extracted.

Our target was the V3–V4 hypervariable region of the bacterial 16S rRNA gene. PCR was started immediately after the DNA was extracted. The 16S rRNA V3–V4 amplicon was amplified using a 2× Hieff^®^ Robust PCR Master Mix (Yeasen, 10105ES03, Shanghai, China). Two universal bacterial 16S rRNA gene amplicon PCR primers (PAGE purified) were used: the amplicon PCR forward primer (CCTACGGGNGGCWGCAG) and the amplicon PCR reverse primer (GACTACHVGGGTATCTAATCC). The reaction was set up as follows: microbial DNA (10 ng/µL) 2 µL; amplicon PCR forward primer (10 µM) 1 µL; amplicon PCR reverse primer (10 µM) 1 µL; 2× Hieff^®^ Robust PCR Master Mix (Yeasen, 10105ES03, China) (total 30 µL). The plate was sealed and PCR was performed in a thermal instrument (Applied Biosystems 9700, Waltham, MA, USA) using the following program: 1 cycle of denaturing at 95 °C for 3 min, first 5 cycles of denaturing at 95 °C for 30 s, annealing at 45 °C for 30 s, elongation at 72 °C for 30 s, then 20 cycles of denaturing at 95 °C for 30 s, annealing at 55 °C for 30 s, elongation at 72 °C for 30 s, and a final extension at 72 °C for 5 min. The PCR products were checked using electrophoresis in 2% (*w*/*v*) agarose gels in TBE buffer (Tris, boric acid, EDTA) stained with ethidium bromide (EB) and visualized under UV light.

##### Library Construction, Quantification, and Sequencing

Hieff NGS™ DNA Selection Beads (Yeasen, 10105ES03, China) were used to purify the free primers and primer dimer species in the amplicon product. Samples were delivered to Sangon BioTech (Shanghai, China) for library construction using the universal Illumina adaptor and index. Before sequencing, the DNA concentration of each PCR product was determined using a Qubit^®^ 4.0 Green double-stranded DNA assay and it was quality-controlled using a bioanalyzer (Agilent 2100, Santa Clara, CA, USA). Depending on coverage needs, all libraries can be pooled for one run. The amplicons from each reaction mixture were pooled in equimolar ratios based on their concentration. Sequencing was performed using the Illumina MiSeq system (Illumina MiSeq, San Diego, CA, USA), according to the manufacturer’s instructions.

##### Sequence Processing, OTU Clustering, Representative Tags Alignment, and Biological Classification

After sequencing, the two short Illumina readings were assembled by PEAR software (version 0.9.8) according to the overlap, and fastq files were processed to generate individual fasta and qual files, which could then be analyzed by standard methods. The effective tags were clustered into operational taxonomic units (OTUs) of ≥97% similarity using Usearch software (version 11.0.667). Chimeric sequences and singleton OTUs (with only one read) were removed, after which the remaining sequences were sorted into each sample based on the OTUs. The tag sequence with the highest abundance was selected as a representative sequence within each cluster. Bacterial and fungal OTU representative sequences were classified taxonomically by blasting against the RDP Database and the UNITE fungal ITS Database, respectively.

##### Statistical Analysis

Based on the results of the OTU clustering analysis, statistical diversity analysis of the sample OTU data was conducted, quantifying *α* diversity indices (including Chao1 and Shannon indices) using OTU richness as a metric. To evaluate sample sufficiency, rarefaction curves of OTU counts were constructed, and *α* diversity indices were calculated using Mothur software (version 1.43.0). *β* diversity was employed to assess differences in microbial communities between samples. Meanwhile, a series of comprehensive statistical analyses and visualizations of community structure at various taxonomic levels were conducted based on OTU clustering information.

The calculation formula for the Chao index is as follows:

$$S_{Chao1}=S_{obs}+\frac{n_{1}(n_{1}-1)}{2(n_{2}+1)}$$

where $S_{Chao1}$ = the estimated number of OTUs; $S_{obs}$ = the observed number of OTUs; $n_{1}$ = the number of OTUs that contain only a single sequence; and $n_{2}$ = the number of OTUs that contain exactly two sequences.

The calculation formula for the Shannon index is as follows:

$$H_{shannon}=-\sum_{i=1}^{S_{obs}}\frac{n_{i}}{N}ln\frac{n_{i}}{N}$$

where $S_{obs}$ = the observed number of OTUs; $n_{i}$ = the number of sequences contained in the *i*-th OTU; and N = the total number of sequences.

##### Function Prediction

FAPROTAX software (version 1.2.1) was used to predict the cycling functions of nutrients such as carbon, nitrogen, and sulfur in soil bacteria. Additionally, functional prediction analysis of bacteria was conducted using PICRUSt (version 2.5.2), which compares existing 16S rRNA gene sequencing data with a database of microbial reference genomes with known metabolic functions, enabling the prediction of bacterial metabolic capabilities.

## 3. Results and Discussion

### 3.1. Characteristics of SDS Degradation in Lake–Terrestrial Ecotone Soil with Different Treatments

**Figure 2:**
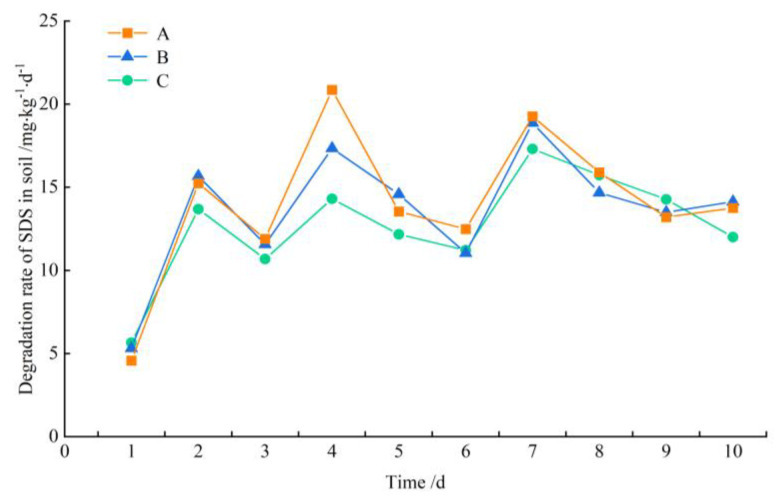
SDS degradation rate in the analyzed experimental variants during the 10 days of the experiment.

According to the experimental analysis, the degradation rate of SDS by soil microorganisms under continuous SDS input at a concentration of 6 mg/L into the lake–terrestrial ecotone is shown in Figure 2. During the first 1~2 days of the experiment, the SDS removal rate increased steadily from 4.57~5.65 mg/kg·d to 13.69~15.67 mg/kg·d, with a 142~234% increase. This increase was attributed to significant changes in the soil bacterial community following SDS stimulation, which enhanced the activity of microorganisms capable of SDS degradation. The degradation rate of SDS fluctuated between 10.69 and 20.87 mg/kg·d during the first 3~7 days of the experiment. It is speculated that the bacterial community was undergoing structural changes due to the continuous input of SDS and gradually stabilized. After day 8, the degradation rates in three treatments stabilized, ranging from 12.01 to 15.90 mg/kg·d, indicating that a relatively stable bacterial community had formed in the soil.

Under conditions of long-term and low concentrations of SDS input, the degradation rate of SDS in different types of lake–terrestrial ecotones followed a similar trend, fluctuating between 11 and 16 mg/kg·d, with the rates in descending order being A > B > C. An analysis of variance (ANOVA) revealed no significant difference in degradation rates among the treatments (*p* > 0.05), suggesting that the growth status of aquatic plants did not significantly influence the ability of soil microorganisms to degrade SDS in the lake–terrestrial ecotone.

### 3.2. Processes of Microorganisms’ Response to SDS Contamination in the Lake–Terrestrial Ecotone with Different Treatments

#### 3.2.1. Effect of SDS on Bacterial Community Species Diversity

**Figure 3:**
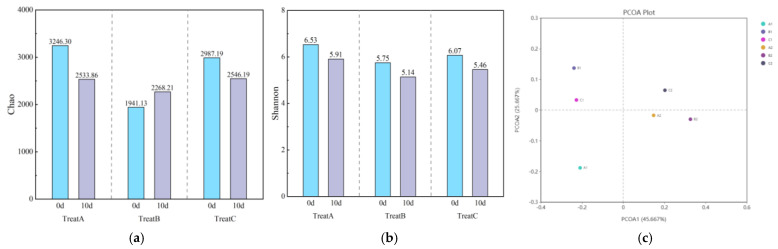
Histograms illustrating the changes in Chao (**a**) and Shannon (**b**) indices, along with a PCoA analysis plot (**c**), across various treatments during the 10-day experimental period.

Bacterial diversity is commonly examined in community ecology, often through *α* diversity analysis of soil samples, which reflects both species richness and the degree of uncertainty (diversity) within the bacterial community. As shown in Figure 3a,b, when the SDS concentration in the environment was maintained at 6 mg/L, changes in the bacterial diversity index in the lake–terrestrial ecotone, corresponding to different aquatic plant conditions, exhibited a specific pattern. In particular, the Chao index (Figure 3a) of the lake–terrestrial ecotones with healthy (A) and severely damaged (C) aquatic plant conditions decreased from 3246.30 to 2533.86 and from 2987.19 to 2546.19, respectively, representing declines of 21.95% and 14.76% following continuous SDS input. Conversely, in the environment with minor damage to aquatic plants (B), the Chao index increased from 1941.13 to 2268.21, reflecting a 16.85% rise after continuous SDS input. Experimental results indicate that in the lake–terrestrial ecotone with minor damage to aquatic plants (B), the small increase in the Chao index suggests a rise in bacterial species, consistent with the results from similar environments exposed to low concentrations of SDS in a single input. In contrast, in environments with healthy (A) and severely damaged (C) aquatic plants, long-term exposure to a constant SDS concentration led to a significant decline in bacterial species, with predominantly negative impacts on community diversity.

The Shannon index (Figure 3b) reflects the uncertainty within a bacterial community, representing its level of diversity. Results indicate that the Shannon index of soil microorganisms in all three treatments exhibited a decreasing trend after long-term exposure to low concentrations of SDS over 10 days. In the lake–terrestrial ecotone with varying aquatic plant conditions, the decrease in the Shannon index was similar, ranging from 5.75~6.53 to 5.14~5.91, representing a reduction of 9.50~10.56%. This result suggests that continuous SDS input consistently led to a decline in the diversity of soil bacterial communities. It is speculated that the stress caused by low concentrations of SDS promotes the dominance of bacterial species capable of tolerating and degrading SDS, thereby exacerbating the uneven distribution of species within the community.

The results of the *β* diversity PCoA analysis (Figure 3c) indicate that the first principal coordinate (PCoA1) and the second principal coordinate (PCoA2) explain 45.667% and 25.667% of the variance in sample composition differences, respectively, suggesting that the analysis effectively captures the differences among samples. From Figure 3c, it is evident that there are significant differences in species composition before and after the addition of SDS. Specifically, samples B1 and C1 exhibit more similar species compositions before the addition of SDS. After SDS was introduced into the riparian zone system, the species composition differences among samples A2, B2, and C2 decreased, resulting in increased similarity.

#### 3.2.2. Effect of SDS on Soil Microbial Biomass Carbon and Nitrogen

**Figure 4:**
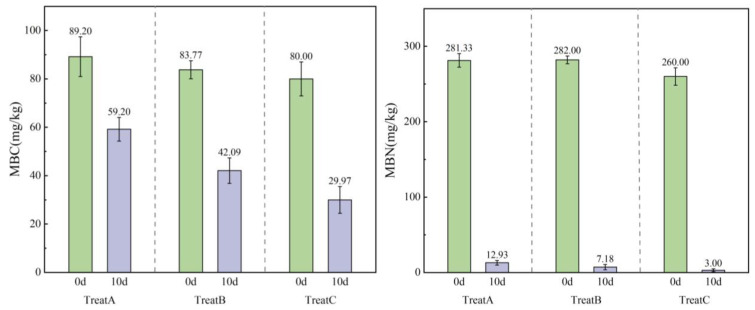
Histograms illustrating the changes in soil microbial biomass carbon and nitrogen content across various treatments during the 10-day experimental period.

Soil microorganisms play a crucial role in nutrient cycling and balance, and their biomass serves as a key indicator for assessing soil health and fertility. Microbial biomass carbon (MBC) and nitrogen (MBN) are effective indicators of soil microbial biomass. As shown in Figure 4, stress from long-term and low concentrations of SDS caused a significant decrease in MBC content across the different treatment groups, with an average reduction of 48.64%. An analysis of variance (ANOVA) revealed significant differences in soil MBC between treatment groups before and after SDS contamination (*p* < 0.05), though no significant differences were observed among groups at the same time points (*p* > 0.05). Similarly, after 10 days, the MBN content in the soil of the lake–terrestrial ecotone decreased significantly compared to that of day 0, with an average reduction of over 95%. Experimental results showed that long-term and low-concentration SDS pollution had more pronounced negative effects on the biomass of soil microorganisms in the lake–terrestrial ecotone, particularly in treatments with poorer aquatic plant growth (B and C), which experienced larger decreases. This suggests that long-term SDS contamination may reduce microbial activity in the lake–terrestrial ecotone, particularly in areas with poor aquatic plant conditions, thereby weakening soil material cycling and energy flow processes.

#### 3.2.3. Effects of SDS on Bacterial Community Structure

**Figure 5:**
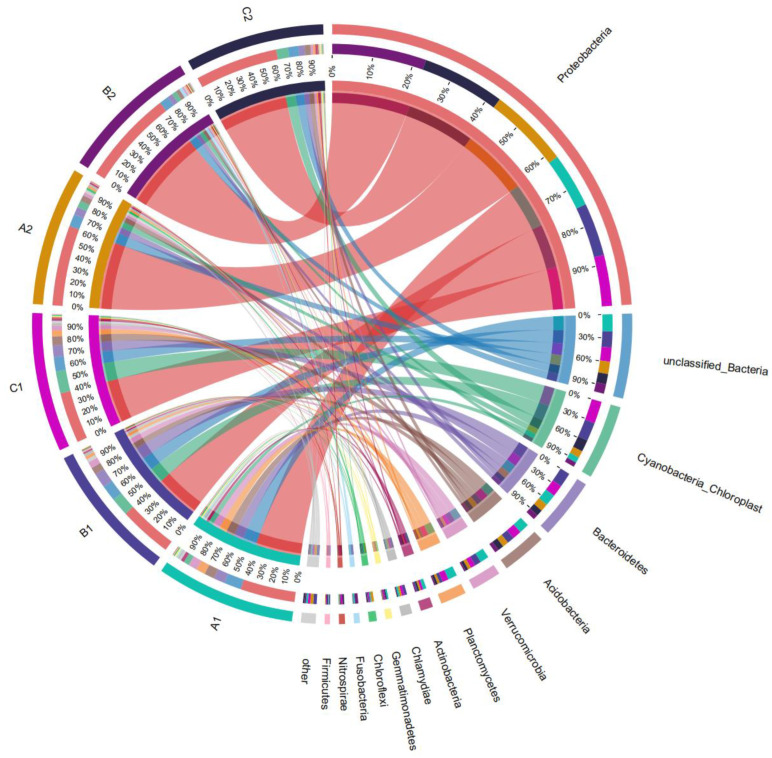
Collinearity diagram of the distribution of the main phylum of the lake–terrestrial ecotone bacterial community in the simulation experiment.

Following high-throughput sequencing analysis, the collinearity distribution of the major phyla of soil microorganisms in the lake–terrestrial ecotone is illustrated in Figure 5. *Verrucomicrobia* was the dominant phylum across lake–terrestrial ecotone environments in all treatment groups, and its relative abundance did not vary significantly among the soils prior to SDS contamination. Following long-term and low concentrations of SDS contamination, the relative abundance of *Proteobacteria* increased from 39.06~41.95% to 61.69~72.43%, marking a substantial rise. By the end of the experiment, the highest relative abundance of *Proteobacteria* was observed in the treatment with minor damage to aquatic plants (B2), while little difference was found between the treatments with well-grown and severely damaged aquatic plants (A2 and C2). The *α* diversity index of the bacterial community was low in lake–terrestrial ecotones where the relative abundance of *Proteobacteria* was high (TB). This aligns with findings from previous studies and further confirms that long-term exposure to low concentrations of SDS can homogenize the soil bacterial community structure. The likely cause is that contamination promotes the excessive dominance of *Proteobacteria* within the community, thereby disrupting bacterial stability and impairing material cycling functions in the lake–terrestrial ecotone.

Similarly, the long-term and low-concentration SDS inputs had a non-negligible effect on changes in the relative abundance of other dominant microorganisms in the soil bacterial community of the lake–terrestrial ecotone. *Verrucomicrobia* is an important organic matter-degrading bacterium commonly found in a variety of soil and freshwater environments [16]. Studies have shown that *Verrucomicrobia* has the ability to degrade and utilize a wide range of complex carbohydrates, and also participates in a variety of biogeochemical cycling processes such as the soil carbon cycle and sulfur cycle [17]. The experimental results showed that the relative abundance of *Verrucomicrobia* in all three treatments decreased more significantly from 0 to 10 days, following the order B > A > C. Correlation analysis revealed that the relative abundance of *Verrucomicrobia* was significantly negatively correlated with *Proteobacteria* (*p* < 0.05), and significantly positively correlated with the bacterial community richness index (Shannon index) (*p* < 0.05). Similarly, *Actinobacteria* can drive the transformation of organic matter in the environment and play an important role in organic matter decomposition [18]. Especially under harsh conditions, *Actinobacteria* can produce a variety of bioactive molecules, which in turn promote the oxidation and decomposition of pollutants [19]. Under the influence of long-term and low-concentration SDS pollution, the relative abundance of *Actinobacteria* in all three treatments showed a certain degree of decrease, ranging from 18.86 to 47.97%. *Planctomycetes* is widely known as an important participant in the nitrogen cycle and is found mainly in freshwater [20], marine sediments [21], soils [22], and in other anaerobic environments. All bacteria with anaerobic ammonia oxidation functions identified so far are from the phylum *Planctomycetes*, which also plays an important role in biological nitrogen removal in wastewater [23] and soil nitrogen removal [24]. Studies have shown that *Planctomycetes* responds rapidly to changes in the concentration of SDS in the environment [12]. The relative abundance of *Planctomycetes* in all treatments decreased significantly after the low concentrations of SDS stress was maintained for at least 10 days, ranging from 56.40 to 74.51% compared with the initial level.

**Figure 6:**
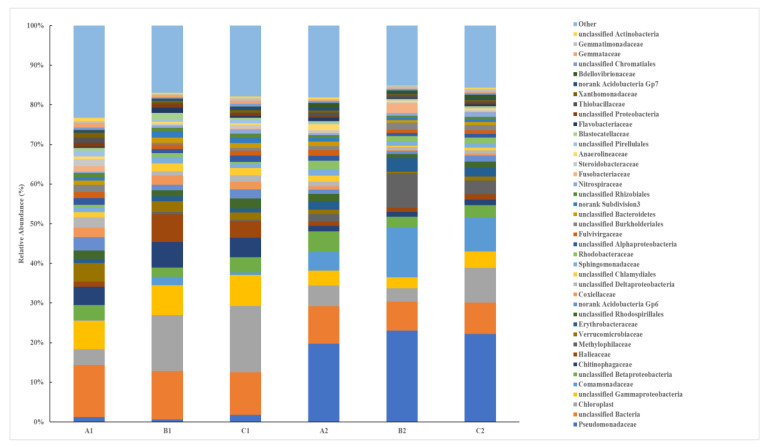
Distribution histogram of the main families of soil microorganisms in the lake–terrestrial ecotone.

At the family level of microbial taxonomy, prior to the introduction of SDS into the lakeshore system, the major dominant families included *Chloroplast*, *Gammaproteobacteria*, *Chitinophagaceae*, and *Halieaceae* (Figure 6). However, after a sustained low concentrations of SDS was continuously introduced into the system over a period of 10 days, significant alterations in the microbial community structure were observed. *Pseudomonadaceae*, *Chloroplast*, *Gammaproteobacteria*, *Comamonadaceae*, and *Methylophilaceae* emerged as the dominant classes. Notably, *Pseudomonadaceae* in all three treatments occupied an absolute dominant position in the microbial community, with the average relative abundance increasing from 1.20% to 21.67%. Various microbes identified as possessing SDS degradation capabilities belong to *Pseudomonadaceae*, indicating that the long-term introduction of low-concentration SDS stimulated the growth of SDS-degrading bacteria, thereby accelerating the degradation of SDS in the environment [12]. *Comamonadaceae* increased from 1.01% prior to the introduction of SDS to 8.63%. Since *Comamonadaceae* is closely related to the denitrification process [25], this suggests that the introduction of SDS also altered the nitrogen cycling processes involving soil microbes. Meanwhile, the relative abundance of *Chloroplast* and *Halieaceae* exhibited a decreasing trend compared to levels observed prior to the introduction of SDS, declining by 50.32% and 71.03%, respectively. Research indicates that *Halieaceae* plays a significant role in metal detoxification and carbon fixation-related processes [26]. The decline in its relative abundance may also indicate that the carbon cycling processes of soil microbes were impacted by the long-term stress of SDS.

**Figure 7:**
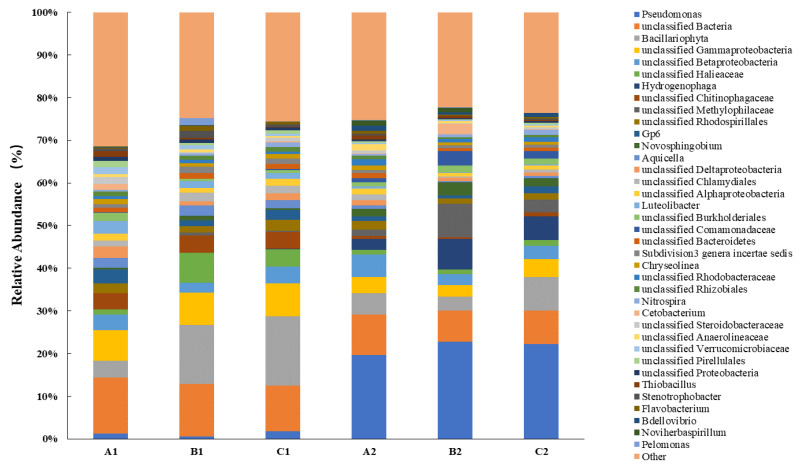
Distribution histogram of the main genera of soil microorganisms in the lake–terrestrial ecotone.

At the genus level of microbial taxonomy, differences in bacterial community structure were observed in samples from the three treatment groups prior to the introduction of SDS into the lake–terrestrial ecotone (Figure 7). *Bacillariophyta*, *Betaproteobacteria*, *Gammaproteobacteria*, *Chitinophagaceae*, and *Gp6* exhibited high relative abundance in the bacterial community structure of the A1 samples and were the dominant genera in the community. In contrast, the bacterial community structures of samples B1 and C1 displayed notable similarities, with *Bacillariophyta*, *Gammaproteobacteria*, *Halieaceae*, and *Chitinophagaceae* identified as the dominant genera. After 10 days of continuous SDS input, the soil bacterial community underwent significant changes. The relative abundance of *Pseudomonas*, a well-known SDS-degrading bacterium, increased substantially. In the newly formed bacterial community, the relative abundance of *Pseudomonas* rose from 0.61~1.78% to 19.65~22.77%, becoming the dominant genus. This suggests that under SDS stress, the proportion of degrading bacteria in the bacterial community increased, facilitating the rapid removal of organic pollutants (SDS) in the lake–terrestrial ecotone. Similarly, two other genera with increased abundance in the treatment group, *Methylophilaceae* and *Comamonadaceae*, are also known for their potential to degrade organic pollutants. *Hydrogenophaga*, a representative denitrifying bacterium, exhibited a very low relative abundance (<0.04%) in the soil bacterial community of the lake–terrestrial ecotone. The relative abundance of *Hydrogenophaga* increased 160-fold following SDS stress. Its relative abundance was significantly higher in treatments with fewer aquatic plants (B2 and C2) compared to treatments with healthy aquatic plant conditions (A2). This indicates that the distribution of this microorganism may be influenced by the presence of aquatic plants.

Notably, the relative abundance of *Acidobacteria* subgroup *Gp 6* significantly decreased in all treatments exposed to long-term SDS stress. This change markedly differed from the effects of short-term contamination with SDS. Zhu et al. [11] indicated that the relative abundance of *Gp 6* in soil initially decreased following a single addition of 6 mg/L of SDS solution, subsequently increasing dramatically as SDS was degraded. This discrepancy arose because the activity of *Gp 6* was persistently suppressed as the SDS concentration in the soil remained stable. The relative abundance of *Acidobacteria* in soil exhibits a negative correlation with soil organic carbon (SOC) and a positive correlation with soil pH. Consequently, the decrease in the relative abundance of *Gp 6* may indicate a decline in pH, as well as diminished organic matter inputs to the treatment soils throughout the experiment. A rapid increase in the relative abundance of *Chitinophaga* was observed following a brief period of SDS contamination. *Chitinophaga* typically increases only when the bacterial community encounters an inhospitable environment and can induce other bacteria to exhibit antagonistic traits against pollutants. Under the influence of long-term SDS contamination, the relative abundance of *Chitinophaga* declined by 75~88%. Similarly, *Aquicella* and *Luteolibacter* exhibited novel variations. The increased abundance of *Aquicella*, which harbors several pathogenic microorganisms, serves as evidence that SDS contamination enhances pathogenic bacterial activity in natural waters in the short term. However, under continuous SDS input, the relative abundance of *Aquicella* was maintained at low levels. This suggests that the activity of this genus in soil primarily increases during the re-degradation phase, following a decrease in SDS concentration. *Luteolibacter* is primarily located in the inter-root zones of plants and plays a crucial role in the carbon cycle of lakes. The relative abundance of *Luteolibacter* decreased by over 70% when SDS concentrations in the environment were maintained at a constant level. This trend contrasts with the pattern of decreasing and subsequently increasing *Luteolibacter* levels following a single addition of SDS.

#### 3.2.4. Functional Gene Abundance in Microorganisms

According to the experimental results (Table 2), the primary carbon cycle functional genes identified were chemoheterotrophy, aerobic-chemoheterotrophy, chloroplasts, and photoheterotrophy. After 10 days of low-concentration SDS stress, the gene abundance of aerobic-chemoheterotrophy increased from 22.91~37.46% to 41.09~43.88% across all treatment groups, indicating a greater contribution of aerobic-chemoheterotrophy during SDS degradation and utilization. In contrast, the gene abundance of chloroplasts decreased from 15.41~49.87% to 4.13~11.34% prior to the experiment. This suggests that long-term and low concentrations of SDS input may alter the primary carbon cycling pathways of soil microorganisms in the lake–terrestrial ecotone. Regarding changes in carbon cycle functional genes, the treatment group with minor damage to aquatic plants (B1) exhibited the lowest expression of these genes before the experiment began. After exposure to SDS for up to 10 days, the predicted values of carbon cycle functional genes in this treatment group (B2) showed no significant difference from the initial values. This indicates that carbon cycle functional genes in the lake–terrestrial ecotone with minor damage to aquatic plants were less affected by long-term and low concentrations of SDS. This phenomenon contrasts with the effects observed from a single SDS input, where treatments with well-growing aquatic plants experienced less impact. It is hypothesized that in the treatment group with minor damage to aquatic plants, the maintained low concentrations of SDS provided a relatively stable carbon source for the microbiota involved in the carbon cycle, enabling these microorganisms to alleviate environmental stress caused by long-term and low concentrations of SDS. At the conclusion of the experiment, carbon cycle gene abundance in treatments with well-growing aquatic plants (A2) and severely damaged aquatic plants (C2) decreased significantly by 58.23% and 54.41%, respectively, compared to the initial values. Ultimately, the expression levels of carbon cycle functional genes across the three different treatments showed no significant differences. This further indicates that the overall effect of long-term and low-concentration SDS input on the carbon cycle in any lake–terrestrial ecotone environment is consistent.

Prior to SDS input, the treatment with severe damage to aquatic plants (C1) exhibited the highest relative abundance of bacterial nitrogen-fixation functional genes. Following long-term exposure to low concentrations of SDS, the results indicated a decreasing trend in bacterial nitrogen-fixation functional genes across different treatments, measuring 43.53% (A2), 40.00% (B2), and 82.67% (C2) of the initial values, respectively. This contrasts with the effects observed from a single SDS input, which exhibited varying trends in nitrogen-fixation functional genes across treatments. It is speculated that long-term and low concentrations of SDS damaged nitrogen-fixation bacteria, hindering recovery to initial levels and ultimately reducing the nitrogen-fixing capacity in the lake–terrestrial ecotone across treatments. Changes in nitrification functional genes were positively correlated with alterations in nitrogen-fixation functional genes. In contrast, the changes in functional genes related to nitrogen-respiration exhibited an opposing trend, with the functional genes in the three treatments significantly exceeding initial values at the conclusion of the experiment, averaging an increase of 434.33%.

Under long-term exposure to low concentrations of SDS, sulfur cycle functional genes exhibited a significant decrease, averaging 68.38%. This indicates that the response of sulfur cycle functional genes to long-term and low concentrations of SDS in the lake–terrestrial ecotone across different treatments was consistent and independent of the ecotone’s health status. In contrast, a single SDS input resulted in a significant increase in sulfur cycle functional genes, highlighting the negative impact of long-term and low concentrations of SDS on the sulfur cycle within the lake–terrestrial ecotone.

Furthermore, the abundance of functional genes associated with human pathogens exhibited a substantial reduction due to long-term exposure to low concentrations of SDS. Research on short-term SDS contamination indicates that when SDS is degraded by microorganisms, the gene abundance of human pathogens in newly formed bacterial communities may recover to or even exceed initial levels. In contrast, microorganisms possessing functional genes associated with human pathogens and animal parasites will remain continuously suppressed if SDS concentrations in the environment are sustained over an extended period.

The results of functional gene prediction indicate that genes involved in carbon, nitrogen, and sulfur cycles were significantly suppressed in environments with long-term SDS contamination. As an organic fungicide, SDS possesses both bacteriostatic properties and carbon source provision, resulting in dual effects on microorganisms. Sudden or short-term SDS contamination may stimulate the activation of certain functional genes in microorganisms, including nitrogen respiration and sulfate respiration functional genes. Simultaneously, as SDS disrupts the stability of bacterial communities, pathogenic bacteria may proliferate during the community recovery process. Long-term and low concentrations of SDS induced a transformation in the soil bacterial community, severely impairing the functions of carbon, nitrogen, and sulfur cycling while effectively inhibiting activity against soil pathogenic bacteria.

**Figure 8:**
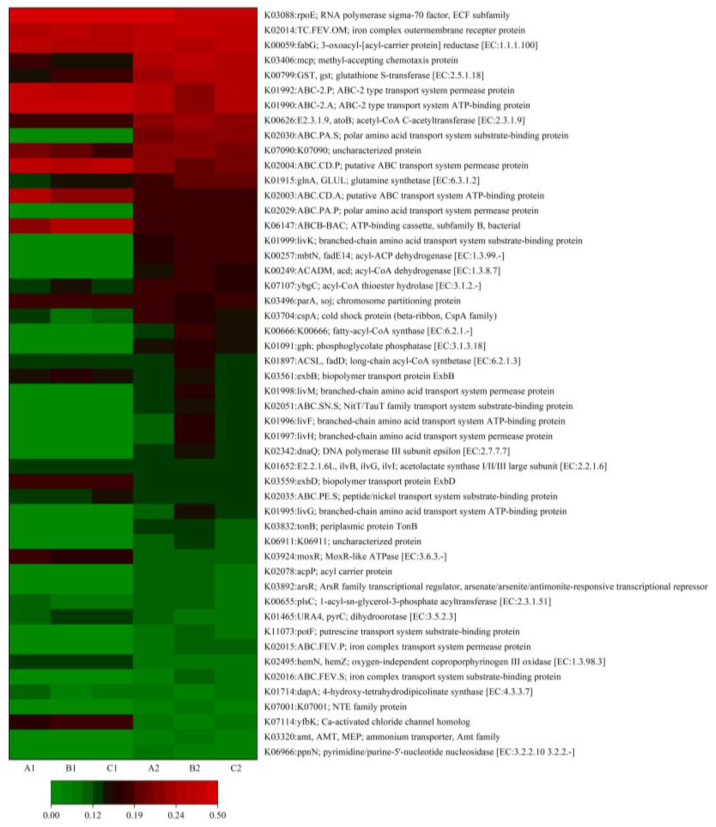
Heat map of KO metabolic pathways in soil microorganisms under long-term and low-concentration SDS input condition.

To further elucidate the effects of long-term low concentrations of SDS contamination on cellular metabolic pathways in soil bacterial communities, we categorized the molecular functions of genes by ortholog groups and KEGG Orthology (KO), identifying changes in functional composition using the KEGG pathway database (PICRUSt, version 2.5.2). As shown in Figure 8, the metabolic pathways with the greatest variation in relative abundance following long-term input of low concentrations of SDS were the methyl-tolerant chemotaxis protein (K03406) and the SGPS-associated gene (K00799). K03406 is a relevant gene that aids microbes in mitigating toxicity from environmental pollutants, such as heavy metals. Previous studies indicated that short-term SDS contamination resulted in a slight decrease in the abundance of K03406, suggesting that microbes may experience reduced stress from environmental pollutants. However, after long-term SDS exposure, the abundance of K03406 increased dramatically, indicating that the source of survival pressure on soil microorganisms shifted with extended SDS contamination. Further supporting this inference was the significant increase in polar amino acid transport system substrate-binding protein (K02030). Both K03406 and K02030 are part of the bacterial chemotaxis signaling pathway, which enables bacteria to accumulate in optimal environments for growth [27] by regulating the rotation of the bacterial flagellum [28]. Gabel et al. [29] proposed that cells sense changes in surrounding chemical signals through the MCP and execute biased motility with the aid of the flagellum and TfP to locate suitable environments for survival. Given the close link between bacterial chemotaxis genes for specific pollutants and degradation genes, an increase in chemotaxis-related genes may indicate significant proliferation of SDS-degrading bacteria [30]. Bacteria may enhance the degradation of metabolizable pollutants through chemotaxis [31], increasing opportunities for degrading bacteria to locate suitable carbon, nitrogen, and other nutrients, thereby maintaining (or even gaining) an advantage in nutrient competition with indigenous microorganisms. This is especially crucial for the bioremediation of low-concentration pollutants [32]. Another metabolic pathway that exhibited a significant increase in abundance was acetyl-CoA C-acetyltransferase (K00626). K00626 is a relevant gene in carbon metabolism, primarily involved in the ethyl malonyl pathway, hydroxybutyrate–dicarboxylate cycle, and hydroxypropionate–hydroxybutyrate cycle. However, the trends of KO metabolic pathways were largely consistent across the different vegetation sites of the lake–terrestrial ecotone, differing primarily in the amplitude of change.

Zhu et al. [12] investigated the effects of short-duration, single-occurrence SDS pollution on microorganisms in the lake–terrestrial ecotone, typically resulting from rainfall runoff that carries pollutants into lakes or rivers. When small amounts of sewage containing low concentrations of SDS enter the lake–terrestrial ecotone, most are degraded by microorganisms within 96 h; however, the impact on the soil bacterial community is not entirely mitigated after 14 days [12]. If SDS contamination persists for an extended period, soil microorganisms are compelled to transform into a new community structure [33]. The effects of this transformation on bacterial species and gene function differ significantly from those of episodic SDS contamination. In this scenario, a subset of bacteria with SDS tolerance and degradation capacity, such as *Pseudomonas*, assumes a dominant role, leading to a decrease in the *α* diversity index of the soil bacterial community in the lake–terrestrial ecotone. The new bacterial community exhibits a higher SDS degradation capacity of 11~16 mg/kg·d, influenced by soil properties, temperature, and other contaminants. Altering the status and species of aquatic plants in the lake–terrestrial ecotone had minimal impact on SDS degradation rate; however, soil bacterial biomass was highest in treatments with healthier aquatic plants, likely because most microorganisms promoted by these plants (e.g., *Firmicutes*) lack the capacity to degrade SDS [34]. Regarding the functional genes of microorganisms, episodic SDS contamination may lead to a temporary increase in the abundance of human pathogen genes [11]. Zhu et al. [35] investigated the effects of SDS on microorganisms on the surface of gangue and proposed that SDS alters the structure of the original microbial community, leading to an increased risk of pathogenicity over a short period. This change persists for more than 21 days. However, this phenomenon does not occur as long as SDS stress persists in the environment, indicating that the activation of pathogenic bacteria takes place during the bacterial community recovery phase after SDS degradation. The primary impact of long-term and low concentrations of SDS pollution on the lake–terrestrial ecotone environment is evident in substance cycling, with a significant decrease in the abundance of genes related to nitrogen, sulfur, and some carbon cycles. Such changes may severely hinder the lake–terrestrial ecotone’s ability to purify pollutants. In terms of metabolic pathways, the new bacterial community not only exhibits enhanced tolerance to and decomposition of organic pollutants but also demonstrates increased chemotaxis. Bacteria facilitate the degradation of metabolizable pollutants through chemotaxis, a phenomenon that has been frequently reported in studies of other organic pollutants. Adadevoh et al. [36], in their study of chemotaxis and the degradation of *Pseudomonas putida G7* on naphthalene, found that bacterial chemotaxis enhanced its motility in naphthalene-contaminated soil, which, in turn, significantly improved naphthalene degradation efficiency. Chemotaxis can be classified into two types: positive and negative chemotaxis. Positive chemotaxis aids microorganisms in locating available substrates, whereas negative chemotaxis directs microorganisms to evade toxic and harmful substances [36]. Krell et al. [30] found that bacteria exhibit chemotaxis for most of their degradation substrates, thereby demonstrating a close link between chemotactic genes and degradation genes for specific pollutants. Harwood et al. [37] and Hawkins [38] also found direct molecular evidence that degradation genes for a variety of pollutants are co-regulated by plasmids, as are chemotactic genes. As is SDS preferentially adsorbed from the water column onto the surfaces of soil particles and exhibits uneven distribution in the environment, the enhancement of chemotaxis represents a targeted strategy for microorganisms to respond to pollution [39]. To mitigate the impact of SDS-containing effluent on the lake–terrestrial ecotone, increasing the area of wetlands is an effective strategy. Expanding wetland areas serves as an effective approach to mitigating the effects of SDS-containing wastewater on the riparian zone. For instance, a small sewage treatment plant with discharge of 1000 t/a would require approximately 3200 m^2^ of wetland to adsorb and degrade the anionic surfactants in its effluent. Other substrates to which microorganisms can attach may also fulfill similar functions [40,41].

## 4. Conclusions

In this study, a combination of a large-scale simulation pool and a custom-designed simulation device was employed to conduct experiments, revealing a series of effects caused by long-term and low concentrations of SDS on soil bacterial communities within the lake–terrestrial ecotone. However, due to limitations in the experimental conditions, the findings require further validation in a natural lakeshore zone environment.

(1) Under long-term and low concentrations of SDS stress, the degradation rate of SDS by soil microorganisms in the lake–terrestrial ecotone ranged from 11 to 16 mg/kg·d. Modifying the vegetation status of the lake–terrestrial ecotone did not significantly influence the SDS degradation rate. The bacterial adaptation period occurred within 1 to 7 days post-pollution, with the SDS degradation rate gradually stabilizing after 8 days.

(2) Long-term and low concentrations of SDS stress significantly altered the bacterial community structure within the lake–terrestrial ecotone. In addition to *Pseudomonas*, the relative abundance of organic pollutant-degrading bacteria, including *Methylophilaceae* and *Comamonadaceae*, as well as denitrifying bacteria such as *Hydrogenophaga*, increased significantly. Conversely, the activity of genera such as *Gp 6* and *Chitinophaga* was notably inhibited. These alterations are significantly different from the environmental impacts associated with short-term SDS pollution.

(3) Long-term and low-concentration SDS pollution resulted in the significant impairment of gene functions related to nitrogen, sulfur, and aspects of the carbon cycle in microorganisms of the lake–terrestrial ecotone, while also partially inhibiting pathogenic bacteria in the soil. After 10 days of continuous pollution, soil microorganisms displayed enhanced chemotaxis, regarded as the primary strategy for coping with long-term SDS stress.

## Figures and Tables

**Table 1:** Habitat conditions in different simulated pools of the lake–terrestrial ecotone.

Treatments	Status of Aquatic Plants	Hygrophytes (*Lythrum salicaria*)	Emerging Plant (*Acorus calamus*)	Submerged Plants (*Vallisneria natans*)
T A	Aquatic plants grow well	12 plants/m^2^	16 plants/m^2^	24 plants/m^2^
T B	Minor damage to aquatic plants	6 plants/m^2^	10 plants/m^2^	15 plants/m^2^
T C	Severe damage to aquatic plants	0	4 plants/m^2^	6 plants/m^2^

**Table 2:** Prediction of functional genes of bacterial communities in lake–terrestrial ecotone using FAPROTAX (version 1.2.1) database.

Functional Genes		A1	B1	C1	A2	B2	C2
Carbon cycle	hydrocarbon_degradation	165	32	100	5	0	0
	chitinolysis	1205	74	465	102	46	32
	chloroplasts	6885	8812	23,685	1458	942	2455
	chemoheterotrophy	21,059	7038	12,855	8971	12,480	10,115
	methanotrophy	165	26	100	5	0	0
	methanol_oxidation	63	405	433	495	2279	911
	methylotrophy	228	431	533	500	2279	911
Nitrogen cycle	aerobic_nitrite_oxidation	575	404	1507	122	169	305
	nitrification	613	412	1520	123	171	311
	nitrogen_fixation	170	275	352	74	110	291
	nitrate_respiration	31	86	76	267	303	393
	nitrate_reduction	207	158	393	390	398	464
	nitrogen_respiration	33	86	89	267	303	393
	ureolysis	371	5	171	100	136	138
Sulfur cycle	sulfate_respiration	223	518	90	178	51	48
	sulfur_respiration	311	47	94	57	60	12
	sulfite_respiration	5	123	19	7	3	0
	respiration_of_sulfur_compounds	534	565	184	235	111	60
	dark_oxidation_of_sulfur_compounds	12	0	18	3	1	12
Others	human_pathogens	332	153	139	23	23	30
	iron_respiration	342	116	96	61	67	12

## Data Availability

The original contributions presented in the study are included in the article, further inquiries can be directed to the corresponding authors.
